# The network of non-coding RNAs and their molecular targets in breast cancer

**DOI:** 10.1186/s12943-020-01181-x

**Published:** 2020-03-18

**Authors:** Francesca Crudele, Nicoletta Bianchi, Eva Reali, Marco Galasso, Chiara Agnoletto, Stefano Volinia

**Affiliations:** 1 Department of Morphology, Surgery and Experimental Medicine, University of Ferrara, Ferrara, Italy; 2 LTTA, University of Ferrara, Ferrara, Italy; 3 Department of Biomedical Sciences and Specialist Surgery, University of Ferrara, 44121 Ferrara, Italy; 4 IRCCS Istituto Ortopedico Galeazzi, Milan, Italy; 5 Area of Neuroscience, International School for Advanced Studies (SISSA-ISAS), Trieste, Italy

## Abstract

**Background:**

Non-coding RNAs are now recognized as fundamental components of the cellular processes. Non-coding RNAs are composed of different classes, including microRNAs (miRNAs) and long non-coding RNAs (lncRNAs). Their detailed roles in breast cancer are still under scrutiny.

**Main body:**

We systematically reviewed from recent literature the many functional and physical interactions of non-coding RNAs in breast cancer. We used a data driven approach to establish the network of direct, and indirect, interactions. Human curation was essential to de-convolute and critically assess the experimental approaches in the reviewed articles. To enrol the scientific papers in our article cohort, due to the short time span (shorter than 5 years) we considered the journal impact factor rather than the citation number.

The outcome of our work is the formal establishment of different sub-networks composed by non-coding RNAs and coding genes with validated relations in human breast cancer. This review describes in a concise and unbiased fashion the core of our current knowledge on the role of lncRNAs, miRNAs and other non-coding RNAs in breast cancer.

**Conclusions:**

A number of coding/non-coding gene interactions have been investigated in breast cancer during recent years and their full extent is still being established. Here, we have unveiled some of the most important networks embracing those interactions, and described their involvement in cancer development and in its malignant progression.

## Background

The non-coding RNAs are a still growing and heterogeneous set of genes that act upon other non-coding, or coding, RNAs and ultimately regulate most biological processes in the human cell. They have been extensively studied, mainly after year 2000, in human malignancies and particularly in the cancers of the mammary gland.

The studies on non-coding RNAs and breast cancer (BC) prevalently investigate one or few RNAs that have been selected from clinical genomics. Typically, such works analyze the BC transcriptomes from retrospective cohort studies.

**Fig. 1:**
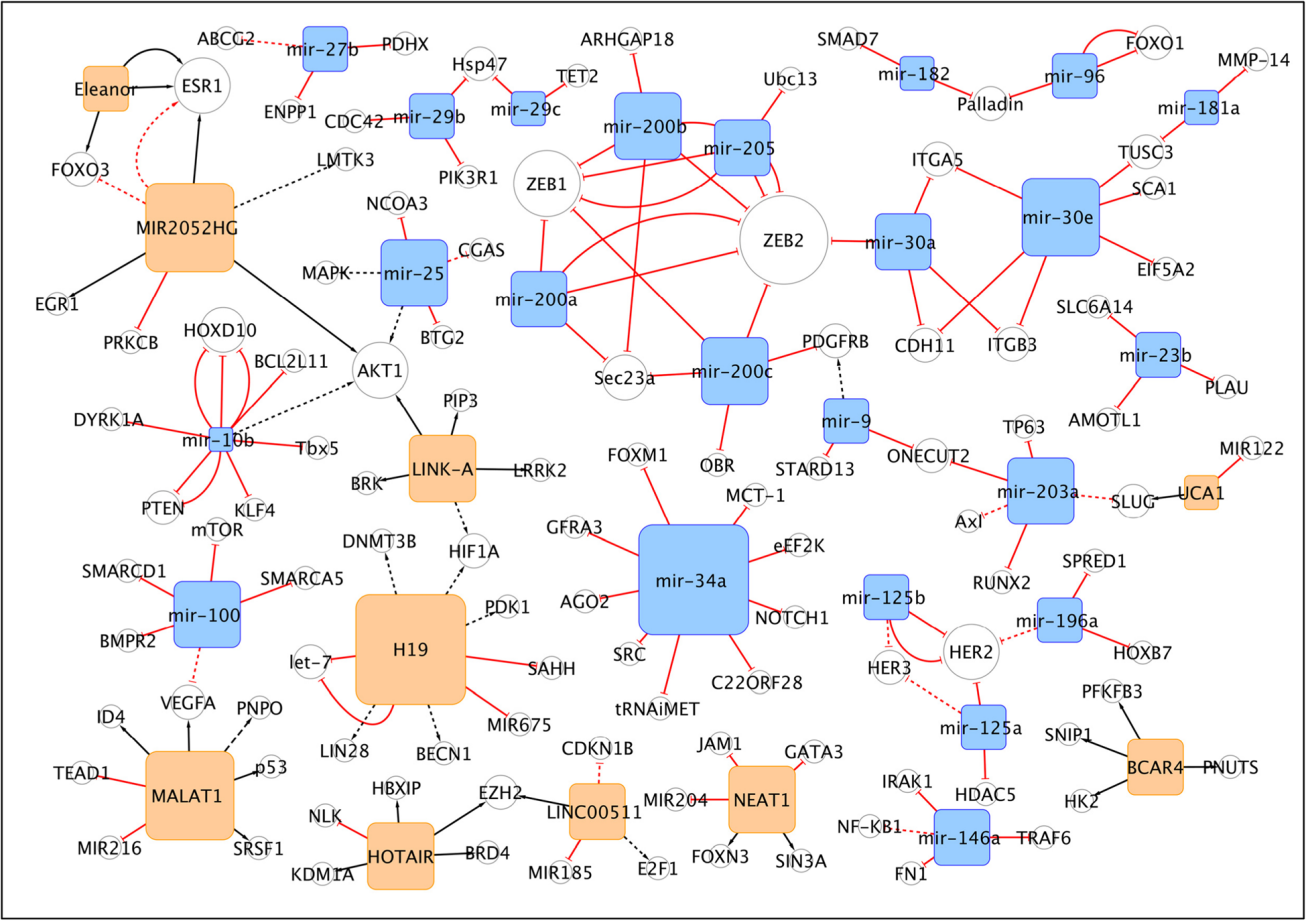
The network of non-coding RNAs and its targets in breast cancer. The graph shows the non-coding RNAs (in the square nodes) cited in at least 2 different sources from literature. Empty circles correspond to the coding genes. Each connecting line (or edge) indicates a publication (PMID) from PubMed. When multiple edges connect the same two RNAs in the network, then multiple publications described this interaction. The edges are directed (i.e. from the non-coding RNA to its target, being either coding or non-coding). In red are depicted the links indicating a repressive action (flat arrowhead), while in black are those showing activation (with traditional arrowhead). Dashed lines correspond to edges indicating indirect effects. The network is the essential core showing what remains after filtering the nodes (non-coding RNAs, in orange, and miRNAs, in light blue) based on their degrees (i.e. the number of connections to targets or other non-coding RNAs). The network’s details are reported in the Table 2

We decided to apply a data-driven study selection rather than use only our human and scientific sensitivity. Firstly, we performed two queries to isolate from PubMed all the articles on ncRNAs and miRNAs published in the last 5 years on BC (Table 1). To triage the studies considered for this review we then selected the journals based on their impact factors. A different, and probably fairer, criterion would have been the citation number, but this is impractical for articles with recent publication time, such as those we wanted to consider here. Furthermore, we let the skeleton of our work to self-assemble using the data themselves. We explored this procedure in our earlier organized view of the role of non-coding RNAs in drug resistance. Using an approach where the nodes are the non-coding RNAs, or their target genes and the edges (connections) are the PMIDs of their relative articles, we obtained a network that was used to organize this review. Separate groups of RNAs and genes that were not linked will be discussed as separate entities or ‘sub-networks’. A statistical analysis of the network helped to identify nodes (RNAs or genes) with particular properties (i.e. degree, or number of interacting RNA/genes) and ultimately for prioritization. The number of citation of an RNA/gene depends both on its ‘real’ importance as determined by the experimental method, or on its ‘perceived’ importance, making it an element of choice by the investigators. The network of non-coding RNAs (ncRNAs) and their targets in BC, defined using this approach is shown in Fig. 1. The graph shows the non-coding RNAs, and their targets, validated in at least two independent sources from literature. The edges are directed (i.e. from the non-coding RNA to its target). In red are depicted the links indicating a repressive action (flat arrowhead), while in black are those showing activation (with traditional arrowhead). Dashed lines correspond to edges indicating indirect effects. The network in Fig. 1 is the essential core showing what remains after filtering the nodes (non-coding RNAs) based on their degrees (i.e. the number of connections to targets). Detailed information about the network composition are reported in Table 2. The filtered out nodes, basically un-replicated findings, are shown in Table 3. They are still worthy of consideration, but were strictly left out of the major network.

**Table 1:** Queries with keywords used for the selection of articles from PubMed

Query	Items found
”Search ((((microRNA OR miRNA OR ncRNA OR ""non coding RNA"" OR lncRNA) AND ""last 5 years""[PDat]) AND (""breast neoplasms""[MeSH Terms] OR ""breast carcinoma"" OR ""breast cancer"")) AND ""last 5 years""[PDat]) Sort by: [pubsolr12]"	5219
”Search (ncRNA OR ""noncodingRNA"" OR ""non coding RNA"" OR lncRNA) AND ""last 5 years""[PDat]) AND (""breast neoplasms""[MeSH Terms] OR ""breast carcinoma"" OR ""breast cancer"")) AND ""last 5 years""[PDat]) Sort by: [pubsolr12]"	4234

**Table 2:** List of ncRNA-target and the type of interaction present in the network

ncRNA	Direct target	Direct effect*	PMID
BCAR4	HK2	pos	28963395
BCAR4	PFKFB3	pos	28963395
BCAR4	PNUTS	pos	25416949
BCAR4	SNIP1	pos	25416949
Eleanor	ESR1	pos	25923108
Eleanor	ESR1	pos	31439835
Eleanor	FOXO3	pos	31439835
H19	DNMT3B	pos	31340867
H19	HIF1A	pos	29106390
H19	let-7	neg	28102845
H19	let-7	neg	29106390
H19	LIN28	pos	28102845
H19	MIR675	neg	30803129
H19	PDK1	pos	29106390
H19	SAHH	neg	31340867
H19	BECN1	pos	31340867
HOTAIR	BRD4	pos	28846832
HOTAIR	EZH2	pos	30764859
HOTAIR	HBXIP	pos	26719542
HOTAIR	KDM1A	pos	26719542
HOTAIR	NLK	neg	30764859
LINC00511	EZH2	pos	31395854
LINC00511	MIR185	neg	30482236
LINC00511	CDKN1B	neg	31395854
LINC00511	E2F1	pos	30482236
LINK-A	AKT1	pos	28218907
LINK-A	BRK	pos	26751287
LINK-A	LRRK2	pos	26751287
LINK-A	HIF1A	pos	26751287
LINK-A	PIP3	pos	28218907
MALAT1	ID4	pos	28652379
MALAT1	MIR216	neg	30982780
MALAT1	p53	pos	28652379
MALAT1	PNPO	pos	30982780
MALAT1	SRSF1	pos	28652379
MALAT1	TEAD1	neg	30349115
MALAT1	VEGFA	pos	28652379
mir-100	BMPR2	neg	25217527
mir-200b	ARHGAP18	neg	28619708
mir-200b	ZEB1	neg	25972084
mir-200b	ZEB2	neg	25972084
mir-200b	Sec23a	neg	25401471
mir-200b	ZEB2	neg	25401471
mir-200c	OBR	neg	25840984
mir-200c	ZEB1	neg	27402080
mir-200c	Sec23a	neg	25401471
mir-200c	ZEB2	neg	25401471
mir-200c	PDGFRB	neg	27402080
mir-203a	Axl	neg	26292363
mir-203a	ONECUT2	neg	31118200
mir-203a	RUNX2	neg	25634212
mir-203a	SLUG	neg	26292363
mir-203a	TP63	neg	26292363
mir-205	Ubc13	neg	25476932
mir-205	ZEB1	neg	25476932
mir-205	ZEB1	neg	26292362
mir-205	ZEB2	neg	26292362
MIR2052HG	EGR1	pos	30944027
MIR2052HG	ESR1	pos	27758888
MIR2052HG	PRKCB	neg	30944027
MIR2052HG	AKT1	pos	27758888
MIR2052HG	FOXO3	neg	27758888
MIR2052HG	ESR1	pos	30944027
MIR2052HG	LMTK3	pos	30944027
mir-23b	AMOTL1	neg	26178901
mir-23b	PLAU	neg	26178901
mir-23b	SLC6A14	neg	31269432
mir-25	BTG2	neg	29310680
mir-25	NCOA3	neg	28920955
mir-25	AKT1	pos	29310680
mir-25	MAPK	pos	29310680
mir-25	CGAS	neg	28920955
mir-27b	ENPP1	neg	26065921
mir-27b	PDHX	neg	30012170
mir-27b	ABCG2	neg	26065921
mir-29b	CDC42	neg	25622979
mir-29b	Hsp47	neg	25744716
mir-100	SMARCA5	neg	25217527
mir-100	SMARCD1	neg	25217527
mir-100	mTOR	neg	28741069
mir-100	VEGFA	neg	28741069
mir-10b	BCL2L11	neg	26359455
mir-10b	DYRK1A	neg	27569213
mir-10b	HOXD10	neg	25428807
mir-10b	HOXD10	neg	26359455
mir-10b	HOXD10	neg	27569213
mir-10b	KLF4	neg	25428807
mir-10b	PTEN	neg	27113763
mir-10b	PTEN	neg	27569213
mir-10b	Tbx5	neg	27569213
mir-10b	AKT1	pos	27113763
mir-125a	HDAC5	neg	25531695
mir-125a	HER2	neg	30068375
mir-125a	HER3	neg	30068375
mir-125b	HER2	neg	25388283
mir-125b	HER2	neg	30068375
mir-125b	HER3	neg	30068375
mir-146a	FN1	neg	30622118
mir-146a	IRAK1	neg	25712342
mir-146a	TRAF6	neg	25712342
mir-146a	NF-KB1	neg	25712342
mir-181a	MMP-14	neg	25977338
mir-181a	TUSC3	neg	28288641
mir-182	Palladin	neg	27641360
mir-182	SMAD7	neg	27996004
mir-196a	HOXB7	neg	26180042
mir-196a	SPRED1	neg	29685157
mir-196a	HER2	neg	26180042
mir-200a	Sec23a	neg	25401471
mir-200a	ZEB1	neg	25972084
mir-200a	ZEB2	neg	25401471
mir-200a	ZEB2	neg	25972084
mir-200b	ARHGAP18	neg	28619708
mir-200b	ZEB1	neg	25972084
mir-200b	ZEB2	neg	25972084
mir-29b	PIK3R1	neg	25622979
mir-29c	Hsp47	neg	25744716
mir-29c	TET2	neg	29109788
mir-30a	CDH11	neg	30042152
mir-30a	ITGA5	neg	30042152
mir-30a	ITGB3	neg	30042152
mir-30a	ZEB2	neg	29666469
mir-30e	CDH11	neg	30042152
mir-30e	EIF5A2	neg	29560860
mir-30e	ITGA5	neg	30042152
mir-30e	ITGB3	neg	30042152
mir-30e	SCA1	neg	29560860
mir-30e	TUSC3	neg	28288641
mir-34a	AGO2	neg	29941603
mir-34a	SRC	neg	26676753
mir-34a	C22ORF28	neg	29187905
mir-34a	eEF2K	neg	29748184
mir-34a	FOXM1	neg	29748184
mir-34a	GFRA3	neg	28356515
mir-34a	MCT-1	neg	30885232
mir-34a	NOTCH1	neg	25368020
mir-34a	tRNAiMET	neg	29941603
mir-9	ONECUT2	neg	31118200
mir-9	STARD13	neg	27402080
mir-9	PDGFRB	pos	27402080
mir-96	FOXO1	neg	27170187
mir-96	FOXO1	neg	29792692
mir-96	Palladin	neg	27641360
NEAT1	FOXN3	pos	28805661
NEAT1	GATA3	neg	28805661
NEAT1	JAM1	neg	25417700
NEAT1	MIR204	neg	30803129
NEAT1	SIN3A	pos	28805661
UCA1	MIR122	neg	29669595
UCA1	SLUG	pos	29774079

*pos positive interaction, activation, *neg* negative interaction, repression

**Table 3:** List of non-codingRNAs, their targets and the type of interactions, cited by only one scientific article, and therefore excluded from the network illustrated in Fig. 1 and further described and discussed in the main text of the review

Non-coding RNA	Target	Type of interaction*	PMID	Reference
AC0269041	SNAI2	pos	29774079	[1]
AGAP2-AS1	MyD88	pos	30157918	[2]
Ai-EGOT	ITPR1, HNRNPH1	pos	30999914	[3]
AK023948	AKT1	pos	28176758	[4]
ANCR	EZH2	neg	27716745	[5]
ARNILA	miR-204	neg	29844570	[6]
ASncmtRNA-1	CCNB1, CCND1, CDK1, CDK4, BIRC5	pos	31142736	[7]
BCYRN1	BCL2L1	pos	27277684	[8]
BORG	RPAIN	pos	30467380	[9]
CASIMO1	SQLE	pos	29765154	[10]
CCAT1	miR-148a/152, miR-204, ANXA2, miR-211	neg	31695775	[11]
circ_0025202	miR-182-5p, FOXO3	neg	31153828	[12]
circAGFG	miR-195-5p	neg	30621700	[13]
circANKS1B	miR-148a, miR-152	neg	30454010	[14]
circEPSTI1	miR-4753, miR-6809	neg	30083277	[15]
circFoxo3	FOXO3	pos	27886165	[16]
circGFRA1	miR-34a	pos/neg	29037220	[17]
circIRAK3	miR-3607	neg	29803789	[18]
circKIF4A	miR-375	neg	30744636	[19]
CYTOR	mTOR, GOLPH3, KIF14, PRKCA, SMYD3	pos	27617288	[20]
DANCR	RXRA, PIK3CA	pos	30518934	[21]
DSCAM-AS1	HNRNPL	pos	27666543	[22]
EFNA3 (NC1 and NC2)	EFNA3	pos	25023702	[23]
ELAV1	CD133	neg	27197265	[24]
EPB41L4A-AS2	RARRES1	pos	30764831	[25]
EPIC1	CDC45, CDC20, CCNA2, CDKN1A	pos	29622465	[26]
Esrp2-as	ESRP2	pos	28759043	[27]
FGF13-AS1	MYCBP, IGF2BP	neg	30771425	[28]
FN1	miR-200c	neg	30967542	[29]
GAS5	miR-222	neg	29969658	[30]
IRAIN	IGF1R	neg	30195750	[31]
LncKLHDC7B	KLHDC7B	pos	30648789	[32]
let-7a	BCL2L1	neg	26915294	[33]
LINC00673	miR-515-5p	neg	31623640	[34]
LINC00968	WNT2	neg	30791958	[35]
LINC01125	LXR-623	pos	30867411	[36]
LINC01133	KLF4	pos	31283068	[37]
LINC01355	CCDN1, FOXO3	neg	31243265	[38]
LINC01638	MYCBP	pos	30002443	[39]
LINC02582	USP7	neg	31601781	[40]
LINCIN	NF90 (a major spliced form of interleukin enhancer binding factor 3, ILF3) CDKN1A	pos, neg	28558830	[41]
LINCK	RSL1D1, ZEB1, ZO-1, CDH1/E-cadherin, CDH2/N-cadherin, VIM	pos, pos, neg, neg, pos, pos	30795783	[42]
LINCRNA-APOC1P1–3	TUBA1A	pos	27228351	[43]
LINC-RoR	DUSP7	neg	29041978	[44]
LINC-ZNF469–3	miR-574-5p	pos	29755127	[45]
LINP1	IGFBP3	pos	30725116	[46]
LncATB	miR-200, TWIST1	neg	30518916	[47]
Lnc-BM	JAK2/STAT	pos	29130936	[48]
lncRNA152, lncRNA67	E2F4	pos	26236012	[49]
lncRNA-Hh	GAS1	pos	26418365	[50]
LOC284454	COL2A1, COL4A1 COL6A1, ITGA2	neg	29227193	[51]
MAYA	MST1, YAP1	pos	28114269	[52]
MBNL1	DBNL, TACC	neg	26883358	[53]
MEG3	TGFB, TGFBR1, SMAD2	neg	26205790	[54]
MIR100HG	CDKN1B	pos	30042378	[55]
miR-101	POMP	neg	26145175	[56]
miR-103, miR-107	NKILA	neg	25759022	[57]
miR-105	MXI1	neg	29588351	[58]
miR-106b	BRMS1L	neg	25406648	[59]
miR-1204	VDR	neg	29555976	[60]
miR-122	PKM	neg	25621950	[61]
miR-124	IL-11	neg	29343249	[62]
miR-1254	CCAR1	neg	27002217	[63]
miR-1285, miR-136	HERC4	neg	30710319	[64]
miR-130a	PTEN	neg	28935812	[65]
miR-132, miR-212	SOX4	neg	26377202	[66]
miR-135	RUNX2	neg	25634212	[67]
miR-135a1	ESR1, ESRRA, NCOA1	neg	29945962	[68]
miR-138	EZH2	neg	25339353	[69]
miR-139	MAT2A, POLQ, TOP1, TOP2A, XRCC5	neg	29180477	[70]
miR-141	TCF12	neg	26068592	[71]
miR-142	APC	neg	25406066	[72]
miR-144	TET2, EIF5A2, ATXN2	neg	29109788	[73]
miR-148a	DKK1	neg	29721077	[74]
miR-148b	ITGA5, ALCAM	neg	27328731	[75]
miR-15	BCL2	neg	26915294	[33]
miR-153	KLF5	neg	26941846	[76]
miR-155	miR-143	pos	26795347	[77]
miR-159	TCF7	neg	26794868	[78]
miR-181c	PDPK1	neg	25828099	[79]
miR-185	E2F1	neg	30482236	[80]
miR-18a	SREBF1	neg	29988076	[81]
miR-190	SMAD2	neg	29510731	[82]
miR-195	ONECUT2	neg	31118200	[83]
miR-199a	LCOR	neg	28530657	[84]
miR-200c/141	HIPK1	neg	30613263	[85]
miR-204	PIK3CB	neg	30737233	[86]
miR-206	TWF1, MAP 3 K9, SPATA6, IL-11	neg, pos, pos, pos	27435395	[87]
miR-20a	MAPK1	neg	29125598	[88]
miR-21	DDX5, PTEN	pos	30413718	[89]
MIR210HG	miR-1226-3p	neg	31399552	[90]
miR-214	miR-148b	neg	27328731	[75]
miR-216b-5p	PNPO	neg	30982780	[91]
miR-218	ZFX	neg	31310241	[92]
miR-221	Beclin1	neg	27940575	[93]
miR-24	ING5	neg	28490335	[94]
miR-31	GNA13	neg	25889182	[95]
miR-320b	NRP1, ETS2	neg	26178901	[96]
miR-329, miR-362	BCAR1	neg	26337669	[97]
miR-33a	ADAM9, ROS1	neg	26507842	[98]
miR-345	KISS1	neg	28981380	[99]
miR-34c	EIF5A2, SCA2	neg	29560860	[100]
miR-3609, miR-5096	CDK1	neg	31142736	[7]
miR-375	TNS3, PXN, CCL2	neg	30850595	[101]
miR-424 (322)/503	BCL2, IGF1R	neg	28404630	[102]
miR-4306	SIX1, CDC42, VEGFA	neg	30867840	[103]
miR-4485-3p	CCNB1, CCND1	neg	31142736	[7]
miR-454-3p	RPRD1A, AXIN2, DKK3, SFRP1	neg	30809286	[104]
miR-4728	ESR1	neg	29476008	[105]
miR-4766-5p	SIRT1	neg	29752439	[106]
miR-484	CDA	neg	25643696	[107]
miR-515	NRAS, MARK4, PIK3CB	neg	26882547	[108]
miR-548a	SIX1	neg	29455928	[109]
miR-548j	Tensin1	neg	26949125	[110]
miR-5582-3p	LUCAT1, TCF7	neg	31300015	[111]
miR-600	SCD1	neg	28249169	[112]
miR-892b	NF-kB, TRAF2, TAB3, TAK1	neg	26747895	[113]
miR-93	NCOA3	neg	28920955	[114]
miR-940	ARHGAP1, FAM134A	neg	29440427	[115]
miR-99a	Her2	neg	25388283	[116]
MPPED2-AS1	DNMT1, MPPED2	neg	31181813	[117]
NAMPT-AS	POU2F2	pos	30940661	[118]
NBR2	AMPK	pos	26999735	[119]
NDRG1-OT1	NDRG1	neg	30497328	[120]
NKILA	Ik-B	neg	25759022	[57]
NONHSAT101069	miR-129-5p	neg	31444414	[121]
NORAD	S100P	neg	30967631	[122]
PDCD4-AS1	PDCD4	pos	30496290	[123]
piR-FTH1	Fth1	neg	30102404	[124]
PIWI-36712	SEPW1P	neg	30636640	[125]
PIWIL3	miR-21, miR-45	neg	28094937	[126]
PNUTS	miR-205, ZEB1, ZEB2	neg, pos, pos	28825698	[127]
PRLB	SIRT1	pos	29752439	[106]
PTENP1	miR-20a, PTEN	neg, pos	31196157	[128]
PTV1	BAP-1, CTNNB1	pos	29760406	[129]
PYCARD-AS1	DNMT1, G9	pos	31086376	[130]
RAINs	RUNX2	pos	28981843	[131]
RP1	p27	neg	31073122	[132]
SNHG5	miR-154-5p	neg	31255976	[133]
SPRY4-IT1	ZNF703	pos	25742952	[134]
ST8SIA6-AS1	PLK1, AURORA	pos	31286138	[135]
T3p	RISC, NUPR1, PANX2	neg, pos, pos	30397354	[136]
TINCR	HER-2, miR-125b, Snail1	Pos, neg, pos	30621694	[137]
TROJAN	ZMYND8, ZNF592	neg	30854423	[138]
WDR7–7	GPR30	pos	29096683	[139]
XIST	c-Met, miR-503	neg, pos	30028120	[140]
YIYA	CDK6, PFKFB3	pos	29967256	[141]

*pos positive interaction, activation, *neg* negative interaction, repression

We will discuss here the most prominent sub-networks and their single components and interactions, with the goal of understanding the involvement and roles of non-coding RNAs in BC.

## The miR-200/205 ZEB2 sub-network

**Fig. 2:**
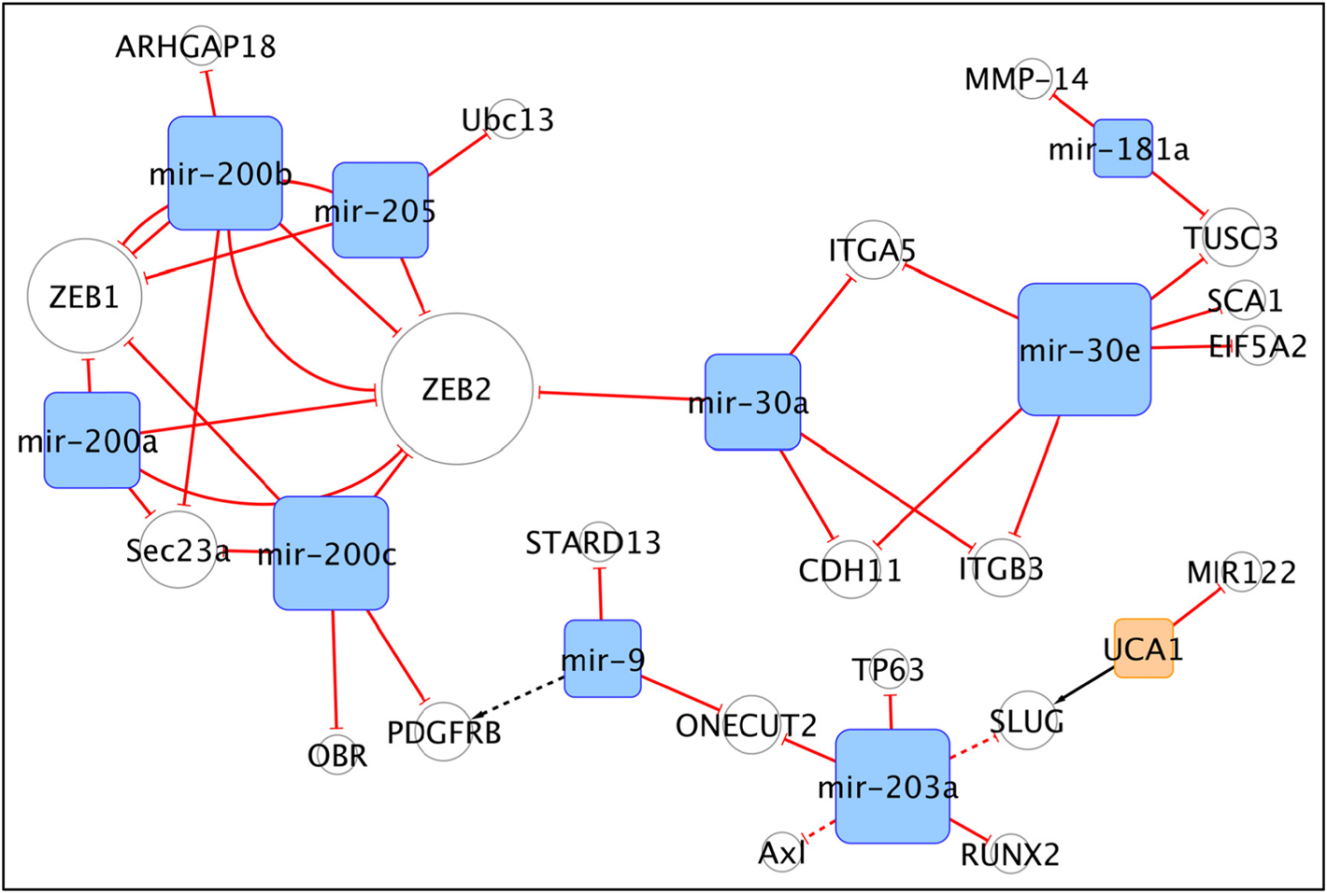
The miR-200 s/ZEB2 cliche

Figure 2 shows that ZEB2 is a pivotal actor in this sub-network, interconnecting the cluster composed by miR-200a/b/c and miR-205 with that of miR-30a/e and miR-181. Several research groups independently asserted that miR-200a/b/c are down-regulated in triple negative breast cancer (TNBC) and function as metastasis suppressor reducing epithelial mesenchymal transition (EMT), tumour invasion and drug resistance [142]. MiR-200 family’s components target other genes that antagonize malignant processes, among them Rho GTPase-activating protein 18 (ARHGAP18), an important regulator of cell shape, spreading, migration, and angiogenesis [143] and the leptin receptor (OBR), which promotes the formation of cancer stem-like cells (CSCs) and up-regulates the obesity-associated adipokine itself associated to BC [144]. Furthermore, in this subnetwork miR-205 is involved in the modulation of basal-like BC motility mediated by the ΔNp63α pathway, by preserving the epithelial cells characters [145]. Mir-205 also is negatively correlated with DNA damage repair, promoting radio-sensitivity in TNBC, by targeting the ubiquitin conjugating enzyme E2N (UBC13) [146]. In contrast, Le et al. demonstrated that delivery of miR-200 family (miR-200a/b/c) by extracellular vesicles, through the circulatory system from highly metastatic tumour cells to poorly metastatic cells, in which ZEB2 and SEC23A were down-regulated, induced EMT and conferred the ability to colonize distant tissues [147]. Further considerations on opposite effects of ncRNAs could be drawn by the second cluster, where the miR-30’s family members suppressed cell invasion in vitro and bone metastasis in vivo by targeting genes implicated in invasiveness (ITGA5, ITGB3) and osteo-mimicry (CDH11) in TNBC [148]. Consistently, miR-30a was involved in EMT regulation, upon TP53 stimulation, by targeting ZEB2 [149]**,** while miR-30e displayed an onco-suppressor role through the modulation of ataxin 1 (SCA1) and EIF5A2, two disruptors of the BC acini morphogenesis promoted by laminin111 (LN1) [100]. MiR-181a could also lead to a reduction in the activation of pro-MMP-2, cell migration and invasion of BC cells through matrix-metalloproteinase MMP-14 [150]. In an apparently opposed fashion, Kuancan et al. demonstrated that miR-181a and miR-30e, once stimulated by SOX2 activation, could promote migration and metastasis dissemination in Basal and Luminal BC via silencing of Tumour Suppressor Candidate 3 (TUSC3) [151]. This subnetwork includes another crucial connection between miR-200c and miR-9, as antagonistic modulators of PDGFR ß-mediated vasculogenesis in TNBC. High levels of miR-9 exerted pro-metastatic function and mediated the acquisition of a mesenchymal and aggressive phenotype. In addition, miR-9 enhanced the generation of vascular lacunae both in vitro and in vivo, in part by direct repression of STARD13, and was also required for PDGFRß-mediated activity. On the other hand, miR-200c in TNBC models strongly inhibited tumour growth and impaired tumour cell–mediated vascularization, by inhibiting PDGFRß activity in vascular lacunae and acting on ZEB1, one of the main transcriptional factors in EMT induction [152]. Furthermore, miR-9 in collaboration with miR-203a could lead to a CSC phenotype and to drug resistance after their release from exosomal vesicles (EV), upon treatment with chemotherapeutic agents. These miRNAs target the transcription factor One Cut Homeobox 2 (ONECUT2), whose reduction induces the expression of a variety of stemness-associated genes, including NOTCH1, SOX9, NANOG, OCT4, and SOX2 [83]. Blocking the EV miRNA-ONECUT2 axis could constitute a potential strategy to maximize the anticancer effects of chemotherapy, as well as to reduce chemoresistance. MiR-203a can collaborate with miR-135 (not showed in this subnetwork) to inhibit cell growth, migration and invasion, by the down-regulation of Runx2 and IL11, MMP-13 and PTHrP targets. Indeed, an aberrant expression of Runx2, which promotes tumour growth and bone metastasis formation, was detected in BC [67]. This subnetwork highlights another connection of miR-203a, occurring with the long non coding UCA1 which affects directly and indirectly the snail family transcriptional repressor 2 (SLUG). MiR-203 prevents the induction of motility in luminal BC cells, through down-regulation of ΔNp63α activity, and the inhibition of its SLUG and AXL targets [145]. Of interest, UCA1 expression in BC cells correlated with TGF-β-induced EMT and tumour metastasis. Mechanistically UCA1 is up-regulated by TGF-β and cooperates with the LINC02599 (AC026904.1) in order to promote SLUG activation and maintenance [1]. Furthermore, UCA1 was proposed to act as a competing endogenous RNA (ceRNA) to sequester miR-122, thus promoting BC invasion. Interestingly, a mechanism mediated by insulin-like growth factor 2 messenger RNA binding protein (IMP1) and repressing invasion has also been hypothesized, via UCA1 decay through the recruitment of the CCR4-NOT1 deadenylase complex. According to this model, IMP1 could compete with UCA1 for binding to miR-122 and restore miRNA targets to inhibit cell invasion [153].

## The LINC0511-HOTAIR subnetwork

The intergenic non-protein coding RNA 00511 (LINC00511) participates in a subnetwork with HOTAIR (HOX transcript antisense RNA), which is linked to the methyltransferase EZH2 and causes impaired cell proliferation and inhibition of apoptosis in estrogen receptor (ER) negative BC cells [154]; indeed, LINC00511 promotes metastasis dissemination by silencing NLK [155]. In this subnetwork LINC00511 was proposed to function as a competitive endogenous RNA, sequestering miR-185, with the effect of inducing E2F1 expression, ultimately leading to stemness and tumorigenesis in all BC subtypes [80]. The other subnetwork member HOTAIR, can act as a scaffold for the late endosomal/lysosomal adaptor, MAPK and MTOR activator 5 (HBXIP), which promotes the expression of three MYC targets, i.e. CCNA1, EIF4E and LDHA, as well as of the lysine demethylase 1A (LSD1), recruited by HBXIP itself [156]. A novel isoform of HOTAIR, named HOTAIR-N, was observed in association with an increase of invasion and metastasis in laminin-rich extracellular matrix-based three-dimensional organotypic cultures (lrECM 3D), compared with traditional “Claudin-low” culture. HOTAIR-N, once cells are attached to extracellular matrix, binds BRD4, a reader of histone markers that recognizes trimethylation on histone H3 lysine 4 [157].

## The H19/LINK-A/MIR2052HG/miR-25/miR-10b/Eleanor sub-network

**Fig. 3:**
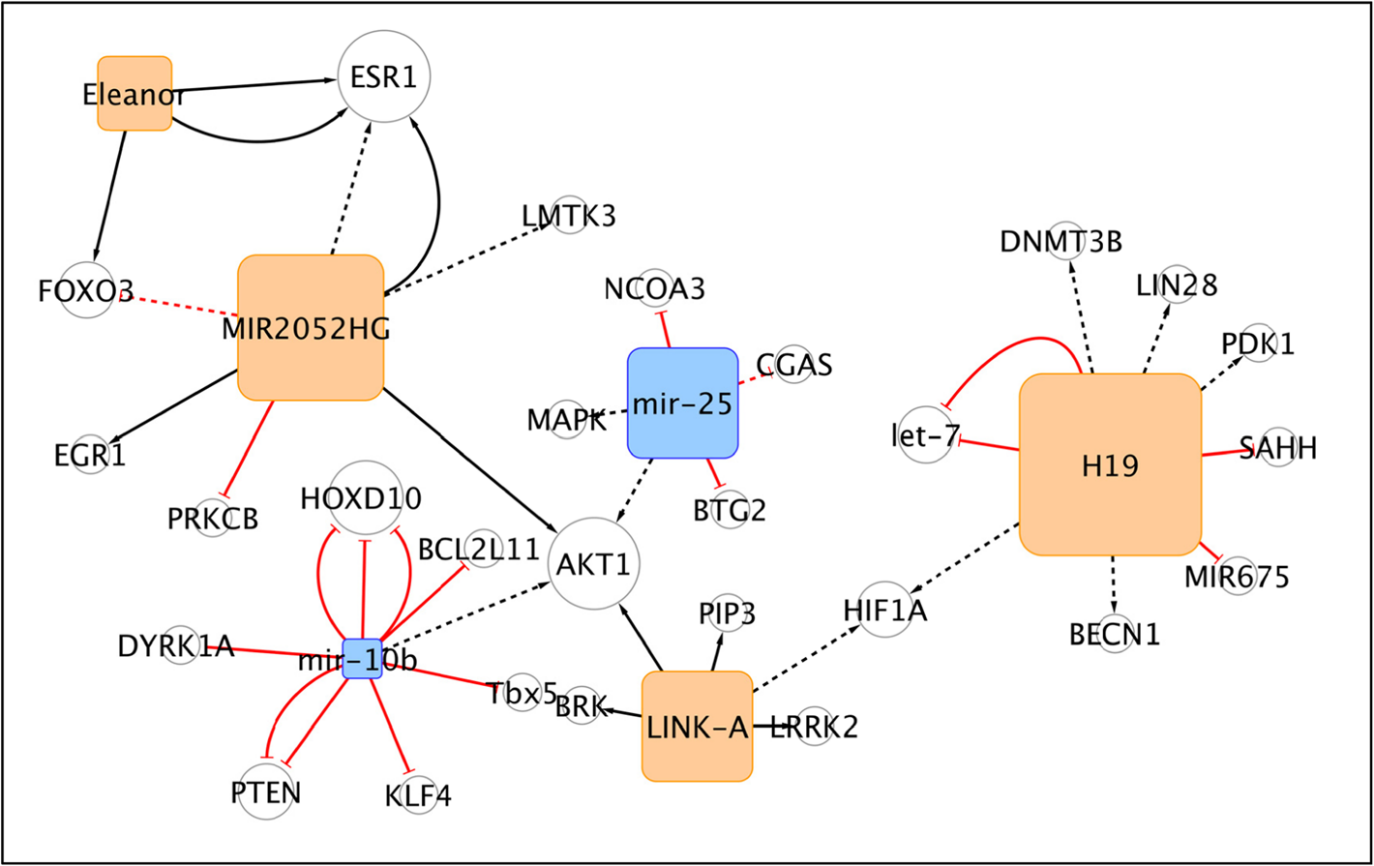
The H19/LINK-A/MIR2052HG/miR-25/miR-10b/Eleanor sub-network

This relatively large sub-network is depicted in Fig. 3. Lnc-H19 and Long intergenic non-coding RNA for kinase activation 01139 (LINK-A) are both indirectly involved in the regulation of the expression of HIF1A. In particular, H19 could induce CSC properties and tumorigenesis possibly via LIN28 by acting as a competitive endogenous RNA towards let-7 miRNA. Furthermore, H19 can indirectly stimulate the expression of HIF1A and PDK1, thus promoting the glycolysis pathway, a crucial step in CSC reprogramming.

H19 and PDK1 therefore may represent possible therapeutic targets, to contrast glycolysis and cancer stem-like properties [158, 159]. Consistently, LINK-A is involved in the normoxic HIF1A stabilization pathway, through the recruitment of the protein tyrosine kinase 6 (BRK) and of LRRK2, that phosphorylate and activate HIF1A itself. From a functional point of view, LINK-A is associated with glycolysis reprogramming in TNBC and promotes tumorigenesis [160]. H19 promotes tamoxifen resistance and autophagy in MCF7 cells, by down-regulating Beclin-1 methylation via epigenetic mechanisms. In details, H19 inhibits adenosylhomocysteinase (SAHH), with subsequent acyl-CoA synthetase medium chain family member 3 (SAH) accumulation, which in turn inhibits Beclin-1 promoter methylation by DNMT3B. Therefore the H19/SAHH/DNMT3B axis was proposed as a therapeutic target against tamoxifen resistance [161]. LINK-A is further connected with MIR2052HG, miR-25 and miR-10b, all known activators of AKT1. In this sub-network a single nucleotide polymorphism (SNP), rs12095274: A > G, in LINK-A affects the phosphorylation status of AKT1 and is associated with AKT inhibitor-resistance by AKT-PREX1 interactions, which results in a worse prognosis for patients [162]. Also MIR2052HG presents a SNP (rs13260300), which have been associated with a higher recurrence of BC and resistance to aromatase inhibitors. MIR2052HG positively regulates estrogen receptor alpha (ERα) via the AKT/FOXO3 pathway, and limiting ERα ubiquitination [163]. MIR2052HG has shown to regulate ERα expression by: *i)* promoting the recruitment of EGR1 on LMTK3 promoter with reduction of PKC activity, indirectly enhancing ERα protein levels; *ii)* limiting ERα ubiquitination via PKC/MEK/ERK/RSK1 pathway. Both mechanisms have been identified as active in the presence of the MIR2052HG SNP rs13260300 and of aromatase inhibitors in ERα-positive BC [164]. MiR-25 can promote cell proliferation in TNBC by silencing B-cell translocation gene 2 (BTG2) and, indirectly, by the activation of AKT and ERK-MAPK pathways [165]. Additionally it has been reported that miR-25 interacts with miR-93 (not present in this network), to down-regulate CGAS, by targeting NCOA3 at its promoter. Hence, it could determine immune evasion and accelerated cell cycle progression under hypoxia in Luminal A cells [114].

The other microRNA engaged in this network is miR-10b which targets HOXD10 and KLF4 to play a pro-oncogenic role. It can promote cell invasion and metastasis formation in the TNBC subtype through its secretion via exosomal vesicles, mediated by neutral sphingomyelin phosphodiesterase 2 (nSMase) indeed and it is capable of transforming non malignant HMLE cells into cells with invasion-ability [166]. Metastasis generation and self-renewal of CSCs driven by miR-10b are the results of the directly inhibition of miRNA target, PTEN, and the indirectly increase of the expression of AKT [167], as well as that of HOXD10 and BCL2 like 11(BIM) [168].

For this reason, miR-10b has been proposed as a “metastamiR”, re-asserted by Kim and co-workers who focused on its targets onco-suppressors Tbx, PTEN, DYRK1A and the anti-metastatic gene HOXD10 [169]. Finally, Eleanor also plays a role in the cluster of non-coding RNAs, cis-activating both ESR1 and FOXO3 [170]. The inhibition of Eleanor could represent a key to switch off topologically associating domain (TAD) containing proteins and to target cells resistant to endocrine therapy [171].

## The MALAT1/miR-100 partnership

**Fig. 4:**
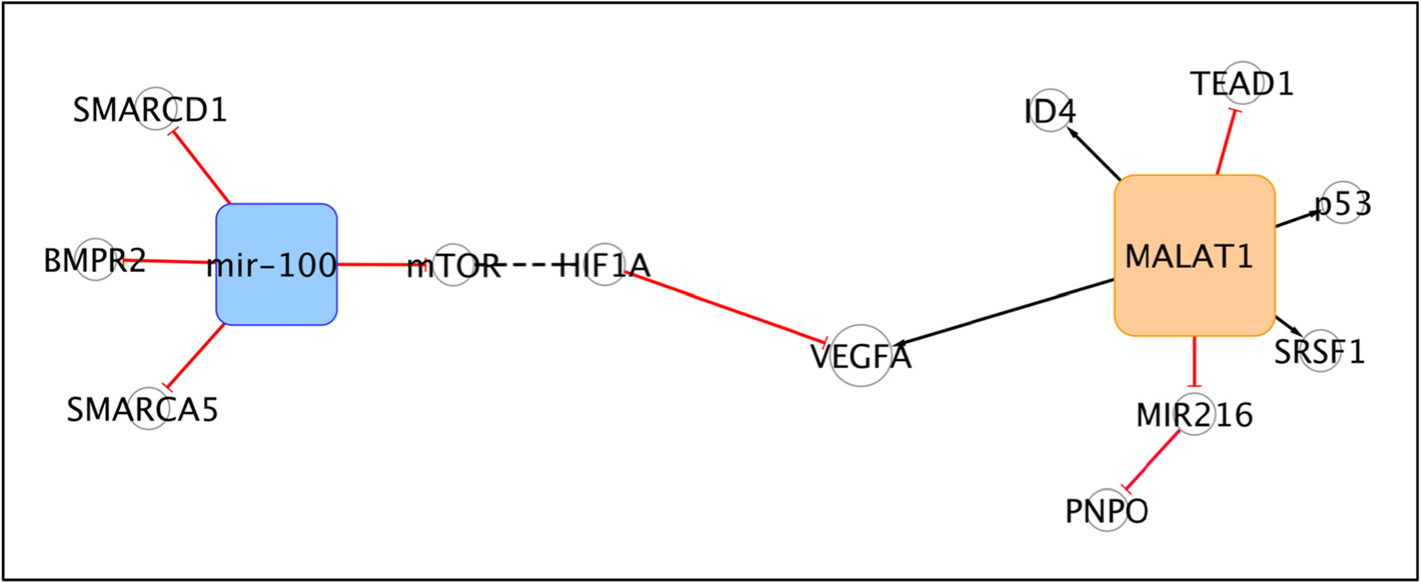
The MALAT1/miR-100 sub-network

The sub-network shown in Fig. 4 evidences long non-coding MALAT1 and miR-100. These non-coding RNAs are indirectly interconnected by VEGFA. MALAT1 modulates VEGFA isoforms expression enhancing TP53 mutations in basal-like BC subtype (BLBC). The interaction between MALAT1 and mutant TP53/ID4 is mediated by SRSF1 splicing factor and promotes MALAT1 delocalization from nuclear speckles and its recruitment on VEGFA pre-mRNA [172].. In addition, MALAT1 acts as competitive endogenous RNA to sponge miR-216b, thus restoring the expression of PNPO, which is associated with promoted cell proliferation, migration and invasion in invasive ductal carcinoma (IDC). MALAT1/miR-216/PNPO pro-metastatic axis represents a target for molecular therapy, as validated in Luminal A and TNBC subtypes [91]. However the role of MALAT1 is still debated. Other studies reported that MALAT1 inhibits the transcription of the pro-metastatic factor TEAD, hindering the interaction between the YAP1 at the TEAD promoters; suggesting MALAT1 as a metastasis-suppressing factor in BLBC [173]. The transfer of miR-100 via MSC-derived exosomes in cancer cells determines the down-regulation of VEGFA secretion by directly targeting mammalian target of rapamycin (mTOR) and modulating mTOR/HIF-1α axis, in fact the miR-100 up-regulation could inhibit angiogenesis and endothelial cell proliferation in the BC microenvironment [174].

Furthermore, mir-100 is negatively correlated with CSC-like self-renewal by inhibiting the SMARCA5, SMARCD1 and BMPR2 regulatory genes in TNBC and Luminal A subtypes. The miR-100 involvement in the inhibition of metastasis has also been validated in vivo [175].

## The miR-125a/b-miR196 sub-network

**Fig. 5:**
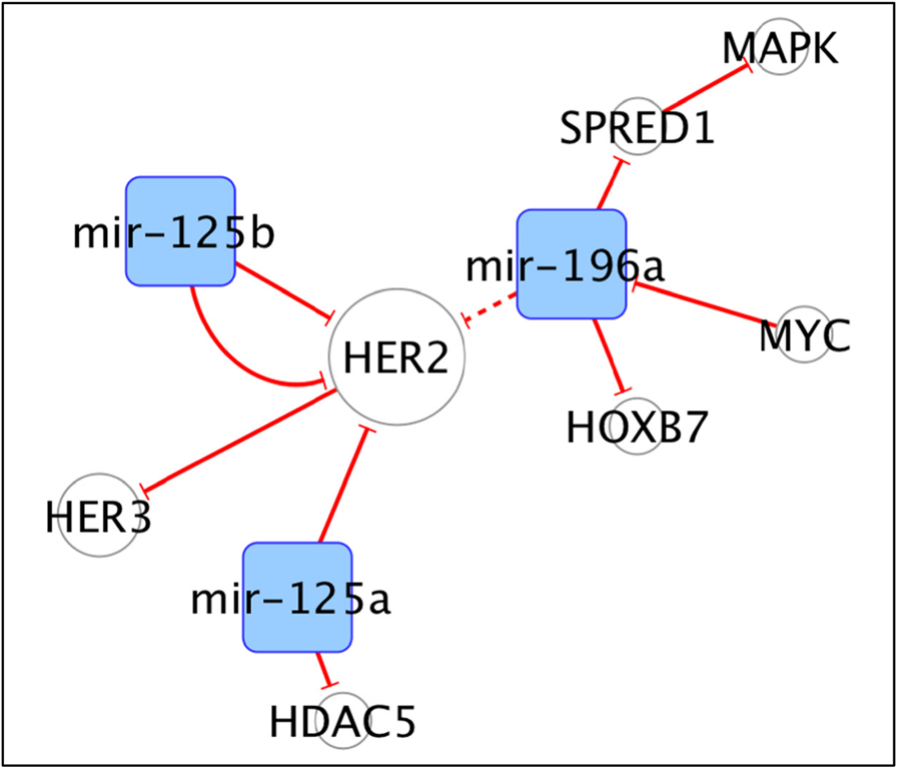
The miR125/HER2 subnetwork

Figure 5 shows the miR-125/HER2 subnetwork. MiR-125a/b target the 3’UTR region of both HER2 which elevates HER3 expression levels, thus reducing HER2 mRNA levels and consequently their oncogenic effects in cellular models, including increase of tumour growth rates and trastuzumab resistance [176]. Consistently, the loss of miR-125b promotes HER2 signalling, and is associated with poor prognosis in patients with Luminal A tumours [116]. MiR-125a exerts also a crucial role in the regulation of apoptosis by silencing of HDAC5, upon stimulation of the RUNX3/p300 pathway, representing a novel anticancer strategy able to activate caspase 3/9 [177]. Indirectly, also miR-196 contributes to inhibit HER2 expression, by altering HOXB7 and HOXB7-ERα interaction. Nevertheless, miR-196 is down-regulated by MYC, which restores HOXB7 and promotes Luminal A breast cancer tumorigenesis and tamoxifen resistance [178]. On the contrary, Jiang et al. demonstrated that miR-196a, upon stimulation by ER-α interaction, promotes growth of Luminal A breast cancer inhibiting SPRED1, a negative regulator of the RAS/RAF/MAPK signalling, indirectly activated by miR-196 [179].

## The miR-182 and miR-96 microRNAs

A study by Yu et al. focuses on the pro-metastatic miR-182, which is associated with EMT, invasion, as well as distant metastasis formation. MiR-182 inhibits the expression of SMAD7, which is both a transcriptional target of TGFβ and a negative regulator of TGFβ signalling [180]. Also, miR-96 modulates the pro-apoptotic FOXO1, a relevant target for precision therapies, and inspired the rational design of TargaprimiR-96 [181]. As a proof of concept, the development of a conjugate small molecule that selectively binds the oncogenic miR-96 hairpin precursor (RIBOTACs), is able to recruit a latent endogenous ribonuclease (RNase L) to FOXO1 transcript, inducing its cleavage. Functionally the silencing of miR-96 de-repressed FOXO1 and induced apoptosis exclusively in TNBC [182]. Other articles highlight an opposite role for these two miRNAs. MiR-96 and miR-182 both target the 3′-UTR region of the PALLD gene. Down-modulation of Palladin transcript expression leads both to decreased migration and invasion of Luminal A breast tumour cells. However, when it is present rs1071738 SNP, a common functional variant of PALLD gene, at the miR-96/miR-182-binding site, the 3’UTR fails to bind the target microRNAs, compromising cell invasion, as verified in in vitro experiments [183].

## miR29b and miR-29c

MiR-29b and miR-29c both target chaperone Hsp47, a modulator of the extracellular matrix (ECM) and promoter of BC development; their indirect regulation of ECM genes reduces collagen and fibronectin deposition [184].

In addition, miR-29c targets TET2, thus inhibiting the metastatic phenotype and the genome instability induced by the conversion of 5-methylcitosine (5-mC) to 5-hydroxymethylcytosine (5-hmC). Nevertheless, in TNBC this condition is antagonized by the lymphoid specific helicase (LSH), which induces miR-29c silencing [73].

Interestingly, miR-29b can act as both inhibitor and promoter of cell proliferation, in Luminal A and TNBC subtypes respectively, based on differential regulation of activation of NFkB and TP53 pathway, mediated by S100A7. In MCF7 cells, S100A7 inhibits NFKB signalling with a consequent upregulation of miR-29b that in turn targets CDC42 and PIK3R1 and indirectly activates TP53 leading to the activation of anti-proliferative pathways. In contrast, in MDA-MB-231 cells, miR-29b which has a lower expression than in MCF7 cells, is suppressed by NFkB with consequent repression of TP53 and promotion of metastasis dissemination [185].

## Other non-coding RNAs relevant in breast cancer

In Fig. 1 we showed all sub-networks, whose ncRNAs have been described in at least two different sources from literature.

One of these ncRNAs is the estrogen-inducible long non-coding NEAT1, which has been proposed to act as ceRNA and ‘sponge’ miR-204. MiR-204 inhibition in turn induced impaired cell proliferation and inhibition of apoptosis. These two processes were supported by the H19 lncRNA [186], to promote para-speckle formation under hypoxia condition, mediated by sequestration of HIF2A and F11 receptor (JAM1) [187]. NEAT1 was also involved in the promotion of invasion, EMT and metastasis dissemination in Luminal A cells by interfering with FOXN3/SIN3A interactions and leading to the repression of GATA3, a crucial regulator of EMT [188].

Another miRNA, miR-27b negatively regulates the acquisition of drug resistance, and is able to induce tumour seeding, two critical properties of CSCs. These effects are mediated by the targeting of ENPP1 and by indirect prevention of the over-expression of ABCG2 transporter. This function was supported by anti-type II diabetes (T2D) drug metformin, that counteracted the generation of CSCs [189]. MiR-27b was also shown to promote the Warburg effect, by inhibiting the PDHX with subsequent dysregulation of the levels of pyruvate, lactate and citrate that increase cell proliferation in the Luminal A and TNBC subtypes [190].

MiR23b has also been subject of recent researches, and itself a notable ncRNA in BC. Its exosome-mediated delivery promoted by Docosahexaenoic acid, an anti-angiogenesis compound, was able to suppress the pro-angiogenic targets PLAU and AMOTL1 in Luminal A and TNBC [96]. Furthermore, in ER-positive endocrine therapy resistant cells, miR-23b was involved in the reprogramming of aminoacid metabolism occurring in association with the down-regulation of SLC6A14 aminoacid transporter, the stimulation of autophagy and the import of aspartate and glutamate by SLC1A2 transporter [191].

The lncRNA breast cancer anti-estrogen resistance 4 (BCAR4) is associated with advanced BC and metastasis. In response to CCL21 chemokine, BCAR4 binds SNIP1 and protein phosphatase 1 regulatory subunit 10 (PNUTS) activating the non-canonical Hedgehog/GLI2 transcriptional program and promoting cell migration [192]. It has been demonstrated that BCAR4 is also involved in the reprogramming of glucose metabolism mediated by YAP1 and favours the transcription of glycolysis promoters HK2 and PFKFB3 via Hedgehog-signalling. The activation of YAP1-BCAR4-glycolisis axis is linked with poor prognosis, and represents an interesting therapeutic target for locked nucleic acids (LNA) delivery, as shown by Zheng et al. [193]

In our review, miR-34a appears as the most discussed non-coding RNA, and several independent research groups all pointed it out as an oncosuppressor. MiR-34a, poorly-expressed in TNBC, revealed its anti-tumorigenic nature by direct targeting of c-SRC [194], GFRA3 [195], and the MCTS1 re-initiation and release factor (MCT-1). Mir-34a also indirectly modulates IL-6, an interleukine associated with breast epithelial acini morphogenesis, and with EMT stimulation in TNBC [196]. Consistently, miR-34a inhibits cancer stem cell properties and promotes doxorubicin sensitivity in MCF7 cells, by targeting NOTCH1. In MCF7 doxorubicin resistant (MCF7/ADR) cells, miR-34a is expressed at low level, possibly due to TP53 mutations [197]. Other effects promoted by miR-34a are the cell-cycle arrest and the apoptosis of TNBC by targeting tRNA_i_^Met^ and AGO2 [198]. Furthermore, miR-34a negatively regulates the EEF2K and FOXM1 proto-oncogenes, both associated with short-term patient survival [199].

The tumour suppressor miR146a, (and its relative miR-146b) is up-regulated by FOXP3 and targets IRAK1 and TRAF6 causing NF-kB inactivation in the Luminal A subtype. The FOXP3/miR-146/NF-kB axis limits tumour growth and could be a valuable target for therapy [200]. The role of miR-146a includes the reduction of fibronectin and opposing to the epithelial phenotype in TNBC subtype with a pro-metastatic activity supported via the oncosuppressor WWOX, that antagonizes MYC functions [201].

## Conclusions

**Fig. 6:**
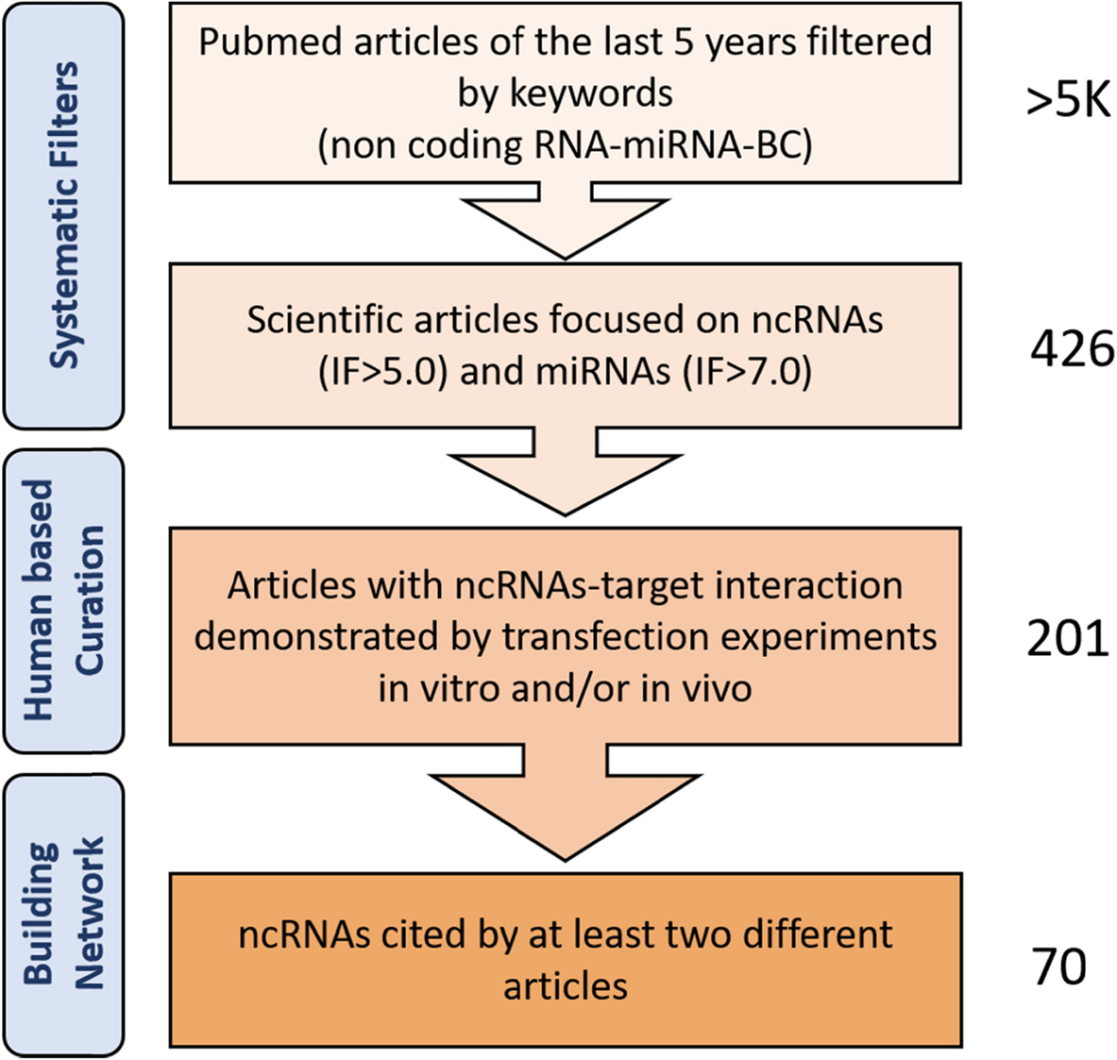
Synthesis of data-approach used to build the network ncRNAs-target. The PubMed queries used for the articles selection are shown in Table 3

The roles of non-coding RNAs in the establishment and evolution of breast cancer are still under scrutiny by many investigators currently active in the field. In this review we performed an unsupervised and large study of the recent literature in the last quinquennium (2014–2019). We used a data-driven approach in order to produce the most unbiased outcome. Orthogonally, we enforced a strict human based curation of each article selection by the PubMed queries. Only papers that clearly applied mechanistic approaches by using in vitro or in vivo methods were included in this review. Thus, we excluded, and did not report, papers with pure correlative analyses, which albeit revealing would not distinguish a causative action of the non-coding RNAs under scrutiny. All steps of our approach are synthesized in Fig. 6.

## Data Availability

not applicable

## Abbreviations

BC: Breast cancer
BIM: BCL2 like 11
BLBC: Basal like breast cancer
BRK: Protein tyrosine kinase 6
ceRNA: Competing endogenous RNA
circRNA: Circular RNA
CSCs: Cancer stem cells
EMT: Epithelial mesenchymal transition
ER: Estrogen receptor
ETR: Endocrine therapy resistant
EV: Exosomal vesicles
HBXIP: Late endosomal/lysosomal adaptorMAPK and MTOR activator 5
IDC: Invasive ductal carcinoma
IMP1: Insulin-like growth factor 2 messenger RNA binding protein
JAM1: F11 receptor
LINCRNA: Large intergenic non-coding RNAs
LN1: Laminin111
lncRNA: Long noncoding RNAs
LSD1: Lysine demethylase 1A
LSH: Helicase lymphoid specific
MCT-1: MCTS1 re-initiation and release factor
miR: Microrna
ncRNA: Non-coding RNA
nSMase: Sphingomyelin phosphodiesterase 2
PIP3: Phosphatidylinositol-3,4,5-trisphosphate dependent Rac exchange factor 1
PNUTS: Protein phosphatase 1 regulatory subunit 10
RNA: Ribonucleic acid
SAH: Acyl-CoA synthetase medium chain family member 3
SAHH: Adenosylhomocysteinase
SCA1: Ataxin 1
SLUG: Snail family transcriptional repressor 2
SNP: Single nucleotide polymorphism
TNBC: Triple negative breast cancer
UBC13: Ubiquitin conjugating enzyme E2 N
